# Immune Cell Response during COVID-19 Infection and following SARS-CoV-2 Vaccination in Patients Admitted to Intensive Care Unit

**DOI:** 10.1155/2023/4059484

**Published:** 2023-04-24

**Authors:** Khadija Bahrini, Nejla Stambouli, Mouna Ben Azaiez, Aicha Rebai, Ferid Abid, Chihebeddine Romdhani, Iheb Labben, Hédi Gharsallah, Mustapha Ferjani

**Affiliations:** ^1^Research Unit UR17DN05, Military Hospital of Tunis, 1008 Montfleury, Tunis, Tunisia; ^2^University Tunis El Manar, Tunis, Tunisia; ^3^Department of Immunology, Military Hospital of Tunis, 1008 Montfleury, Tunis, Tunisia; ^4^Department of Anesthesiology and Intensive Care, Military Hospital of Tunis, 1008 Montfleury, Tunis, Tunisia; ^5^Department of Anesthesiology and Intensive Care, Military Hospital of Gabes, Tunisia

## Abstract

**Background:**

Immune response plays a crucial role in virus clearance during COVID-19 infection and underpins vaccine efficacy. Herein, we aimed to assess the immune response during COVID-19 infection and following SARS-CoV-2 vaccination.

**Methods:**

In this retrospective study, 94 confirmed COVID-19 patients admitted to the intensive care unit were categorized into unvaccinated patients (*n* = 50), including 33 deceased and 17 discharged patients, and vaccinated group (*n* = 44) with 26 deceased and 18 discharged patients. Records of patients with severe COVID-19 admitted to the ICU between March, 2021 and March, 2022 were gathered and analyzed.

**Result:**

The assessment of immune cell counts revealed a large rise of neutrophils associated to decrease number of lymphocytes in patients with COVID-19 infection. In dead patients, we detected a significant correlation between neutrophils and inflammatory parameters such as IL-6 and CRP. Moreover, analysis of immune cell count following vaccination did not reveal any significant difference. However, the most substantial result, herein, detected is the decrease level of IL-6 in vaccinated patients as compared to unvaccinated. The reduce level of IL-6 following vaccination is observed in discharged patients as compared to deceased. Regarding the level of mortality after vaccination, we showed that all patients who received the first dose were died (46.1%, *n* = 12) as compared to those who have received two doses (34.6%, *n* = 9) and the third dose of vaccine (19.23%, *n* = 3) (*p*=0.0018). Strikingly, studying the inflammatory parameters after each vaccine dose, we revealed a significant decrease of IL-6 level after the booster dose (third dose), especially in vaccinated discharged patients.

**Conclusions:**

Neutrophils combined with IL-6 and CRP can be very useful markers to predict disease severity in patients admitted to ICU. The decrease level of IL-6 in vaccinated group pointed out the impact of vaccination to prevent inflammatory cytokine release.

## 1. Introduction

The coronavirus disease 2019 (COVID-19) is a serious pandemic that has caused a substantial mortality and morbidity worldwide. The characteristic of this disease is rather heterogeneous ranging from asymptomatic cases and mild-to-critical illness with severe respiratory failure requiring intensive care [1]. The risk factors for the exacerbation of COVID-19 symptoms from asymptomatic or mild-to-severe disease stages are not well understood. Nevertheless, many reports have suggested that viral genetic variation and evolution might contribute to infectivity and fatality [2, 3]. It is evident that viral genetic variation may not be the sole driving factor that dominates the progression of severe COVID-19 but also immune disturbance seems to correlate closely with disease progression and critical complications [1]. In fact, the innate response with monocytes, dendritic cells, neutrophils, and adaptive immune system with T and B lymphocytes are required to defend against SARS-CoV-2 [4, 5]. However, clinical observations have shown alterations in hematology and immunity in patients with COVID-19 [1, 6]. The increasing number of neutrophils and higher ratio of neutrophil-to-lymphocyte have been reported in most COVID-19 patients [7, 8]. Interestingly, these two clinical characterizations are independent risk factors associated with fatal outcomes [9].

We know so far that vaccination can absolutely reinforce the immune protection against COVID-19. In this context, many studies have demonstrated that SARS-CoV-2 vaccine increases neutralizing antibody responses which provide protection against COVID-19 infection. However, recent study has reported a rapid decline in omicron-specific serum-neutralizing antibody titers only a few weeks after the second and third doses of vaccine [10].

Several reports have evaluated the humoral response and the different subtypes of T cells following vaccination. However, researches which studied the implication of neutrophils and cytokine storm following SARS-CoV-2 vaccination were scarce. For this reason, we aimed to assess the profile of the immune cell population during COVID-19 infection and after SARS-CoV-2 vaccination.

## 2. Materials and Methods

### 2.1. Study Population

**Figure 1:**
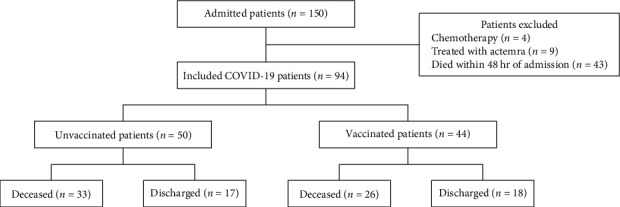
Flowchart depicting patient selection and grouping.

We conducted a retrospective study of 150 patients with confirmed COVID-19 infection upon admission to the intensive care unit (ICU) of Military Hospital of Tunisia (HPMT). The cohort study was implemented from March, 2021 to March, 2022 corresponding to the 5th wave of infection, dominated by the delta and omicron variants. The flowchart showing the selection of the study population is described in Figure 1.

Inclusion criteria were as follows: patients admitted to the ICU with symptoms consistent with COVID-19 and confirmed by RT-PCR. The age of patients enrolled in this study should be more than 18 years.

In this retrospective study, patients who had received chemotherapy before infection (*n* = 4), treated with tocilizumab (interleukin-6 receptor antagonist; Actemra®) (*n* = 9), and died within 48 hr of ICU admission (*n* = 43) were excluded.

Among all the confirmed cases, 94 COVID-19 patients were selected. This cohort study is divided into two groups as follows:

1. Unvaccinated patients (*n* = 50): Among them, 33 patients died and 17 patients discharged.
2. Vaccinated patients (*n* = 44): Including 26 dead patients and 18 discharged.

The disease severity of COVID-19 patients can be divided into four types: mild, moderate, severe, and critical according to the interim guidelines from the WHO and the National Health Commission of China [11]. Patients with slight clinical symptoms and no evidence of pneumonia on computed tomography (CT) imaging were classified as having mild illness. When patients have clinical signs of pneumonia (fever, cough, dyspnea, and fast breathing) but no signs of severe pneumonia, including SpO_2_ ≥ 90% on room air, they are classified as having moderate forms. Patients with severe form of COVID-19 have at least one of the following symptoms: breathing more than 30 times per minute, experiencing severe respiratory distress, or having a room air SpO_2_ below 90% and PaO_2_/FiO_2_ ≤ 300 mm Hg. Critical patients were those who met at least one of the following criteria: they needed mechanical ventilation for their breathing, they showed signs of cardiovascular shock, or they had other organ failures that required monitoring in an ICU. The cases included in this study are therefore in the severe to critical group. This study was approved by the local ethics committee of the Military Principal Hospital of Tunisia (HPMT) (60/2020/CLPP).

### 2.2. Data Collection

For every patient, basic information like history comorbidities, age, sex, smoking, COVID-19 vaccination, and drinking were elaborated through an interview with the patients or families using a standardized questionnaire. Data concerning disease symptoms, dates of onset, diagnosis, hospital admission, discharge, or death were gathered through the hospital electrical records system and inserted into our study database. Daily data such as disease stage, complication, peripheral oxygen saturation (SpO_2_), partial pressure of oxygen in arterial blood (PaO_2_), need for invasive or noninvasive ventilator support, C-reactive protein (CRP) quantification, and clinical impression of patient's evolution were collected. All samples were collected at baseline and throughout hospitalization. Clinical laboratory measurement was gathered at the moment of their admission to ICU. All extracted data were coded and analyzed anonymously.

### 2.3. Clinical Laboratory Measurement of Blood Samples

Peripheral blood was obtained by venipuncture from all the patients admitted to ICU. We have analyzed the immune cell count and the inflammatory parameters such as CRP and the proinflammatory cytokine IL-6. Immune cell count was performed by the hematology analyzer Beckman Coulter DxH 900. CRP and IL-6 analysis were assessed in serum samples using chemiluminescence immunoassay (CLIA) performed on Immulite® 1000, Siemens automate.

### 2.4. Statistical Analysis

All data analysis were performed using Prism software version 5 (GraphPad, San Diego, tailed unpaired CA). Shapiro–Wilk normality test was conducted to estimate the distribution of the data. Categorical variables were reported as percentage. Fisher's exact or *χ*^2^ tests were used to determine their significance. Unpaired *t*-test was used to compare between two groups when continuous variables are normally distributed. Two-tailed unpaired Mann–Whitney *U* test (when comparing two groups), Kruskal–Wallis test, and ANOVA test (for three or more groups) were performed for continuous variables that were not normally distributed. The correlation between neutrophil, IL-6, and CRP was analyzed by Spearman correlation. Differences were considered significant if *p* ≤ 0.05.

## 3. Results

### 3.1. Demographics and Clinical Characteristics of Patients with COVID-19

To better understand the implication of immune system after COVID-19 infection especially following vaccination, we assessed in this current study the amount of immune cell count in dead patients (*n* = 59), discharged (*n* = 35), unvaccinated (*n* = 50), and vaccinated groups (*n* = 44). The demographic and clinical characteristics of these patients are represented in Table 1. The final analysis in this study included 94 adult patients admitted to the ICU with COVID-19 between March, 2021 and March, 2022. The average age of the study population was 67.84 (22–94), 65.82 (24–91) for unvaccinated patients, and 70.14 (22–94) for vaccinated group with a predominance of males in both subgroups. Comorbidities were present in 82 patients (87.23%). The most common was hypertension (60.6%) followed by diabetes (36.17%) and stented coronary artery (10.63%). The vast majority of patients admitted to the ICU died (62.76%) as compared to discharged (37.23%). Only two patients have been previously infected with COVID-19 virus.

All patients received vitamin therapy during their hospital stays in the ICU. The majority of patients (98%) were treated with corticosteroids and antibiotics (96.8%). Every participant enrolled in this research required oxygen therapy.

For vaccinated group, most patients have received Pfizer/BioNTech vaccine (22 patients, 11 dead patients and 11 discharged) and AstraZeneca vaccine (12 patients, 8 deceased and 4 discharged). Additionally, we have six patients who received Sinopharm (5 dead patients and 1 discharged), one patient who received Moderna (discharged), and one patient who had Johnson & Johnson vaccination (deceased). Only the type of vaccine of two patients was not specified. Then, there were 12 patients who received the first dose of vaccine, 23 who received the second dose, and only seven patients who received the third dose of vaccine (Table 2).

### 3.2. Evaluation of Cellular Response in Patients with SARS-CoV-2 Infection

The quantification of different biological parameters in patients admitted to the ICU showed a high activation of leukocytes (21.09 ± 1.29 of the mean) particularly neutrophils (18.2 ± 1.10) in all patients included in this study. Additionally, we revealed a deficiency of lymphocyte (1.32 ± 0.071) and monocyte number count (0.95 ± 0.1).

### 3.3. Peripheral Neutrophil Count Is Strongly Increased in Dead Patients

**Figure 2:**
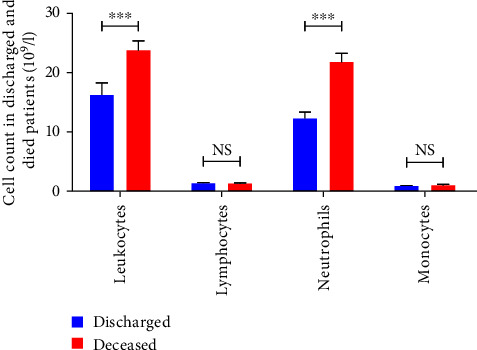
Immune cell count in peripheral blood of all deceased (*n* = 59) and discharged (*n* = 35) patients. Each histogram represents the studied population. Blue boxes correspond to discharged patients and red boxes represent deceased patients. NS, no significant;  ^*∗∗∗*^*p* < 0.001.

We next investigated, in all studied patients, the profile of the different immune cells in dead and discharged groups.  Figure 2 shows a strong infiltration of leukocytes especially neutrophil cells in dead patients as compared to discharged (*p* < 0.0001). However, we have not detected any significant difference concerning the number of peripheral blood lymphocytes and monocytes in both groups (*p* > 0.05).

**Figure 3:**
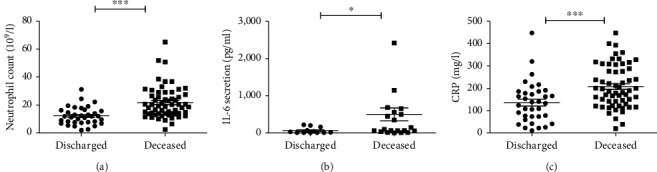
Expression of neutrophil count, IL-6, and CRP level in all deceased and discharged patients: (a) neutrophil count in discharged (*n* = 35) and deceased (*n* = 59) patient; (b) IL-6 expression in discharged (*n* = 33) and deceased (*n* = 34) patients; (c) comparison of CRP level in discharged (*n* = 35) and deceased (*n* = 59) group.  ^*∗*^statistically significant difference; *p* < 0.05 using nonparametric Mann–Whitney *U* test.  ^*∗∗∗*^*p* < 0.001.

We then studied the expression of inflammatory parameters such as IL-6 and CRP in all deceased and discharged patients. Our findings showed a high level of neutrophils, IL-6, and CRP in dead patients as compared to discharged (*p* < 0.0001, *p*=0.02, and *p*=0.0005, respectively) (Figure 3).

**Figure 4:**
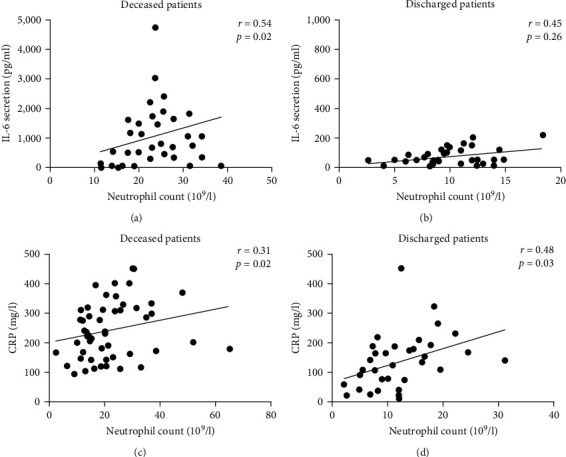
Correlation analysis between neutrophil, IL-6, and CRP in all deceased and discharged patients: (a) a positive significant correlation between IL-6 and neutrophil in deceased patients (*n* = 34, *r* = 0.54, *p*=0.02); (b) there were no association between IL-6 and neutrophil in discharged patients (*n* = 33, *r* = 0.45, *p*=0.25; (c) a positive significant correlation was obtained between CRP and neutrophil (*n* = 59, *r* = 0.31, *p*=0.02) in deceased patients and (d) in discharged (*n* = 35, *r* = 0.48, *p*=0.03) patients.

To better understand the role of neutrophil during COVID-19 infection, we evaluated by correlation analysis, the relationship between neutrophil, IL-6, and CRP. Our result found a significant positive correlation between neutrophil count, IL-6 (*r* = 0.54, *p*=0.02), and CRP (*r* = 0.31, *p*=0.02) in dead patients (Figures 4(a) and 4(c)). However, neutrophil correlates significantly with CRP (*r* = 0.48, *p*=0.03) but not with IL-6 (*r* = 0.45, *p*=0.26) in survivors patients (Figures 4(b) and 4(d)).

### 3.4. Evaluation of Immune Response following SARS-CoV-2 Vaccination

**Figure 5:**
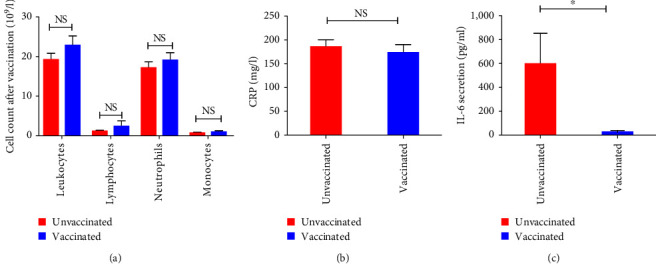
Immune cell count and inflammatory parameters in peripheral blood following vaccination in all studied groups: (a) no significant difference was detected following vaccination; (b) CRP level; (c) IL-6 secretion in unvaccinated and vaccinated patients.  ^*∗*^*p* < 0.05, NS, no significant.

We then analyzed the profile of leukocytes, lymphocytes, neutrophils, and monocytes following SARS-CoV-2 vaccination. Our result did not reveal any significant difference concerning the cell count of different subpopulation in both vaccinated and unvaccinated groups (Figure 5(a)). Interestingly, the assessment of inflammatory parameters following SARS-CoV-2 vaccination showed a significant decrease of IL-6 (*p*=0.03) secretion in vaccinated groups as compared to unvaccinated (Figure 5(c)). However, no significant difference concerning CRP level was detected (*p*=0.33) (Figure 5(b)).

**Figure 6:**
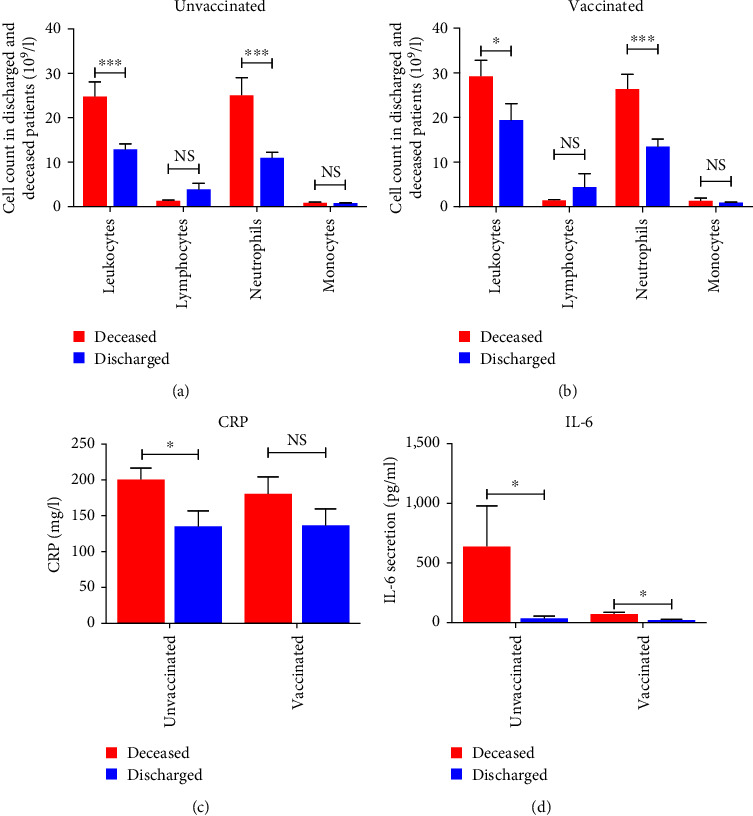
Immune response in unvaccinated and vaccinated patients according to their living status. Unvaccinated deceased patients (*n* = 33), unvaccinated discharged patients (*n* = 17), vaccinated deceased patients (*n* = 26), and vaccinated discharged patients (*n* = 18). NS, no significant;  ^*∗*^*p* < 0.05,  ^*∗∗∗*^*p* < 0.001.

To further understand the impact of vaccination on immune response, we compared the different cell count and inflammatory parameters in deceased and discharged patients considering whether they had received vaccination or not (Figure 6). We found that dead patients have a strong involvement of leukocytes particularly neutrophils count (*p* < 0.001) in both unvaccinated and vaccinated groups (Figures 6(a) and 6(b)). Additionally, our result showed a significant increase in CRP (*p*=0.042) and IL-6 level (*p*=0.027) in unvaccinated deceased patients as compared to unvaccinated discharged patients (Figures 6(c), and 6(d)). The increased level of IL-6 is also detected in vaccinated deceased patients as compared to vaccinated discharged subjects (*p*=0.04) (Figure 6(d)).

On the second hand, we focused on vaccinated patients in order to assess the relationship between the number of vaccine doses and mortality. Our result shows that all patients who received the first dose of vaccine died (46.1%, *n* = 12) as compared to those who received two doses (34.6%, *n* = 9) and the third dose of vaccine (19.23%, *n* = 3) (*p*=0.0018) (Table 2).

**Figure 7:**
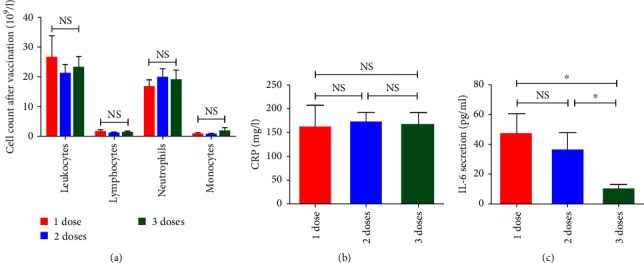
Immune response after the first (*n* = 12), the second (*n* = 23), and the third dose of vaccine (*n* = 7): (a) cell count; (b) CRP level; (c) IL-6 secretion after each dose of vaccine.  ^*∗*^*p* < 0.05, NS, no significant.

To better understand the increased level of mortality after vaccination, we explored the cell response against COVID-19 infection after each vaccine dose in all studied groups (deceased and discharged) (Figure 7). No significant difference concerning the profile of three studied population (lymphocytes, neutrophils, and monocytes) after the first (*p*=0.7), the second (*p*=0.98), and the third dose of vaccine was detected (*p*=0.53) (Figure 7(a)). Evenly, we have not found a significant difference concerning CRP level between the three doses of vaccine (*p*=0.75) (Figure 7(b)). Strikingly, studying the inflammatory parameters after each vaccine dose, we revealed a significant decrease of IL-6 level especially after the booster dose (third dose) (*p*=0.01) (Figure 7(c)). This wane is detected mostly in discharged patients as compared to deceased patients (*p*=0.005) (Table S1).

## 4. Discussion

The assessment of biological parameters in patients admitted to the ICU with COVID-19 infection reveals a prominent signature of neutrophil development, especially in dead patients. Moreover, we demonstrated that vaccination did not induce a large variation in immune cell count during COVID-19 infection. The most striking result revealed in this study was the decreased level of IL-6 in COVID-19 patients following SARS-CoV-2 vaccination.

The host immune response is necessary for infection resistance, but it can also cause costly tissue damage. Neutrophils are the first line of defense against infection and injury, but they can also cause severe collateral damage. A key finding detected in this study is the high neutrophil count associated with a low lymphocyte one with increasing level of IL-6 and CRP in the blood clearly distinguishes severe illness. Lymphopenia detected in COVID-19 patients can be used as a discriminative marker between severe COVID-19 and non-COVID-19 pneumonia [12]. Many studies suggested that the decrease in lymphocyte number mostly caused by T lymphocyte subset depletion, particularly T helper and T suppressor cells [13].

In infectious diseases, neutrophil migrates early to the inflammatory site to eliminate pathogens through phagocytosis and the oxidative burst. In severe SARS-CoV-2 infection, the recruitment and infiltration of neutrophil induce many inflammatory reaction in multiple organs due to the development of neutrophil extracellular traps (NETs) [14]. In this study, we revealed that COVID-19 patients exhibit leukocytosis and neutrophilia, both are correlated to disease severity. This result highlighted the detrimental role of neutrophils in COVID-19. These cells have been observed to be higher in the blood [15, 16] and lungs [17, 18] of severely ill COVID-19. The trafficking of neutrophils to organs such as lung is orchestrated by chemokines. In this context, recent study has demonstrated that neutrophils in patients with moderate disease downregulate CXCR2 expression, potentially limiting their capacity to migrate to inflamed tissues. In contrast, neutrophils in patients with severe disease are characterized by a failure to downregulate CXCR2 expression, indicating maintained capacity for trafficking to organs such as the lungs [19]. Furthermore, the data reported here show that absolute neutrophil count is linked to disease severity which is significantly increased in dead patients. Our observation is in line with other researches which have reported that neutrophil activation signature predicts critical illness and mortality in COVID-19. Markers like neutrophil-derived effectors (RETN), lipocalin-2 (LCN2), and matrix metallopeptidase 8 (MMP8) are among the best predictors of critical illness in COVID-19 provide perhaps the most concrete evidence to date that neutrophil activation is a hallmark of severe disease [20]. Similarly, it has been reported that circulating neutrophil counts are higher in critically ill patient and elevated neutrophil-to-lymphocyte ratios are associated with a worse prognosis which is consistent with our result [21].

Furthermore, the implication of proinflammatory cytokines: interleukin-1*β* (IL-1*β*), IL-2, IL-6, IL-7, IL-8 (CXCL8), IL-9, IL-10, IL-17, IL-18, tumor necrosis factor (TNF-*α*), and interferon-*γ* (IFN-*γ*) in COVID-19 disease is well elucidated. We, herein, focused on IL-6 due to its strong link to disease severity and most importantly it can be exploited as a drug target. The enhanced expression of IL-6 observed in dead patients is consistent with previous report by Cruz et al. [22] who described IL-6 as markedly increased in patients with COVID-19. This cytokine can be used as a biomarker for the development of fatal severe acute respiratory syndrome [22]. Moreover, it has been reported that comorbidities can play a crucial role in immune dysregulation during COVID-19. In this context, obesity and type 2 diabetes both have a characteristic of increased adiposity linked to a persistent, low-grade systemic inflammation that encourages the aberrant production of proinflammatory cytokines such as IL-6 [23, 24]. In addition, we showed that IL-6 levels correlate with neutrophil count in dead patients. This result pointed out the hyperinflammatory status in dead patient as compared to discharged. Across the previous study which revealed that neutrophil and IL-6 can be considered a good prognostic marker for nonsurvivor patients [22, 25]. The increasing count of neutrophil, the decreasing amount of lymphocyte, and the high level of IL-6 and CRP detected in this work may be very useful to predict fatality in patients admitted to the ICU.

Little is known about the implication of neutrophils and inflammatory parameters such as IL-6 after vaccination in patients admitted to the ICU. Interestingly, our findings indicate a significant increase in neutrophil count as compared to lymphocyte and monocyte. However, no significant difference was detected between unvaccinated and vaccinated patients. We also found a similar cellular count after each dose of vaccine in vaccinated group. Based on these results and despite the apparent successful vaccination companies, the immune response in vaccinated patients seems to be unchanged. This finding is in agreement with recent data showing the presence of high antispike IgG titers but a defective immune response in vaccinated patients admitted to the ICU [26]. This data could be explained by genetic variation of COVID-19 virus since its appearance in 2019.

Interestingly, despite the unchanged level of cellular response herein detected, the decreased level of IL-6 in vaccinated group especially in patients who have received the booster dose of vaccine, highlight the protective effect of vaccination against infection. Our findings are concordant with other clinical effectiveness and immunological data showing the benefic effect of a booster dosage and lower infection rates in long-term care facility patients in the time following the introduction of boosters [27, 28].

Nevertheless, despite the fact that vaccine induces a decreased level of IL-6, the mortality rates in vaccinated patients are still greater. Waning of vaccine effectiveness can be associated with many factors. In fact, there are many vaccinated and boosted people are, on average, older (mean age of vaccinated patients is 70.14 years) and having underlying pathologies that put them at risk for severe COVID-19 outcome given the fact that they were in ICU. Additionally, the fading of immune protection against infection over time and low uptake of boosters can be used to explain why there are more dying patients after vaccination. For this reason, the duration and extent of vaccine protection are not well understood especially against newer variants that can partially elude the immune response.

The particularity of this study includes the assessment of cellular immune response among 2–8 months after vaccination rather than just 1–2 weeks later is one of its benefits. Additionally, we compared the cellular response in a larger sample of vaccinated patients than previously reported. Nevertheless, there are several limitations in the current work that should be mentioned. First, because the vaccination campaign started too late in our country (March, 2021), the cohort of patients assessed in this investigation is smaller and needs to be more expanded. Second, this retrospective study limits our research ability to investigate other biological parameters in order to better understand the immunity-boosting effects of COVID-19 vaccination.

## 5. Conclusion

In conclusion, we revealed in this study a high number of neutrophil count and IL-6 in dead patients with COVID-19 infection. These parameters can help clinicians to predict disease severity in patients admitted to the ICU. Moreover, the decreased level of IL-6 herein detected especially in discharged patients and after booster vaccine can be very useful to explain the best outcomes of SARS-CoV-2 vaccination. Therefore, our findings detected in this work may pave the way to other studies in order to assess in-depth the effect of vaccine on immune system and to verify if the vaccination program is an effective strategy to prevent the cytokine storm.

## Figures and Tables

**Table 1:** Patients characteristics.

	Overall patients	Unvaccinated patients	Vaccinated patients	*p*-value
Total, *n* (%)	94 (100%)	50 (53.19%)	44 (46.8%)	0.22
Mean age	67.84	65.82	70.14	0.079
Min–max	22–94	24–91	22–94	
Sex ratio (F/M)	0.67	0.51	0.91	0.2
Female	38	17	21	
Male	56	33	23	
Comorbidities				
Hypertension	57 (60.6%)	29 (30.85%)	28 (29.78%)	0.57
Diabetes	34 (36.17%)	19 (20.21%)	15 (15.95%)	0.83
Chronic kidney disease	8 (8.51%)	6 (6.38%)	2 (2.12%)	0.27
Dyslipidemia	7 (7.44%)	3 (3.19%)	4 (4.25%)	0.7
Chronic lung disease	3 (3.19%)	0	3 (3.19%)	0.098
Cardiovascular disease	8 (8.51%)	2 (2.12%)	6 (6.38%)	0.14
Stented coronary artery	10 (10.63%)	4 (4.25%)	6 (6.38)	0.5
Asthma	4 (4.25%)	2 (2.12%)	2 (2.12%)	–
Stroke	4 (4.25%)	3 (3.19%)	1 (3.19%)	0.62
Neurological disorders	5 (5.31)	2 (2.12%)	3 (3.19%)	0.66
Obesity	3 (3.19%)	1 (1.06%)	2 (2.12%)	0.59
Smoking patients				
Yes	14 (14.89%)	6 (6.38%)	8 (8.50%)	0.56
No	80 (85.1%)	44 (88%)	36 (81.81%)	
History of COVID-19 infection	2 (2.12%)	0	2 (2.12%)	0.21
Deceased	59 (62.76%)	33 (35.1%)	26 (27.65%)	0.52
Discharged	35 (37.23%)	17 (18.08%)	18 (19.14%)	0.52
Treatment				
Vitaminotherapy	94 (100%)	50 (53.19%)	44 (46.8%)	–
Glucocorticoids	92 (98%)	49 (98%)	43 (98%)	0.92
Antibiotic treatment	92 (96.8%)	49 (98%)	43 (98%)	0.92
Oxygen support	94 (100%)	50 (53.19%)	44 (53.19%)	–

**Table 2:** The different types and doses of vaccine.

	Pfizer/BioNTech	Astra Zeneca	Sinopharm	Moderna	Janssen	Total	Deceased	Discharged
Patients (*n*)	22	12	6	1	1	42	24	18
Doses								
1 dose	7	4	0	0	1	12	12	0
2 doses	9	8	5	1	0	23	9	14
3 doses	6	0	1	0	0	7	3	4
Deceased	11	8	5	0	1	–	–	–
Discharged	11	4	1	1	0	–	–	–
COVID-19 infection postvaccination (min–max)	2–8 months							

## Data Availability

All data generated and analyzed in this study are available from the corresponding author on reasonable request.

## Abbreviations

ICU: Intensive care unit
SpO_2_: Peripheral oxygen saturation
PaO_2_: Partial pressure of oxygen in arterial blood
CRP: C-reactive protein
IL: Interleukin
NETs: Neutrophil extracellular traps
RETN: Neutrophil-derived effectors
LCN2: Lipocalin-2
MMP8: Matrix metallopeptidase 8.

## Supplementary Materials

Supplementary Materials Table S1: Immune response after each dose of vaccine in deceased and discharged groups.
