# Supplementary material for: Immune Cell Response during COVID-19 Infection and following SARS-CoV-2 Vaccination in Patients Admitted to Intensive Care Unit
Source: J Immunol Res. 2023 Apr 24;2023:4059484. doi: 10.1155/2023/4059484 (PMC10151725; doi:10.1155/2023/4059484)
Supplement: Supplementary Materials — Table S1: Immune response after each dose of vaccine in deceased and discharged groups. [file 4059484.f1.docx]

**Supplementary data 1**: Immune response after each dose of vaccine in deceased and discharged groups.

|  | Vaccine doses | Deceased(n=24) | Discharged(n=18) | P value |
| --- | --- | --- | --- | --- |
| Leukocytes  Mean(range) | 1 dose | 26.7  (2.70-73.90) | - | 0.15 |
|  | 2doses | 26.09  (7.1-59.70) | 16.01  (9-34.10) |  |
|  | 3dose | 29.16  (12.60-38.40) | 16.10  (9.4-22.80) |  |
| Lymphocytes  Mean(range) | 1 dose | 1.72  (0.6-4.06) | - | 0.82 |
|  | 2doses | 1.29  (0.4-2.7) | 1.2  (0.43-2.24) |  |
|  | 3dose | 1.7  (0.5-2.89) | 1.05  (0.53-1.4) |  |
| Neutrophils  Mean(range) | 1 dose | 25.67  (8.9-73.6) | - | 0.085 |
|  | 2doses | 24.21  (6.5-51.92) | 13.80  (4.9-31.26 |  |
|  | 3dose | 24.56  (9.09-31.36) | 12.45  (7.7-15.68) |  |
| Monocytes  Mean(range) | 1 dose | 1.01  (0.31-2) | - | 0.27 |
|  | 2doses | 0.94  (0.2-1.3) | 0.86  (0.2-2.02) |  |
|  | 3dose | 2.9  (0.72-9.49) | 0.7  (0.36-0.99) |  |
| CRP  Mean(range) | 1 dose | 132.4  (23-360) | - | 0.14 |
|  | 2doses | 207  (20-448) | 132.4  (42-230) |  |
|  | 3dose | 171  (94-309) | 233.8  (106-448) |  |
| IL-6  Mean(range) | 1 dose | 40.35  (14.1-75) | - | 0.0054 |
|  | 2doses | 40.34  (10-75.5) | 13.14  (9.5-18.60) |  |
|  | 3dose | 21.25  (15-25) | 9.37  (6-12) |  |
